# Rapid, continuous projection multi-photon 3D printing enabled by spatiotemporal focusing of femtosecond pulses

**DOI:** 10.1038/s41377-021-00645-z

**Published:** 2021-09-24

**Authors:** Paul Somers, Zihao Liang, Jason E. Johnson, Bryan W. Boudouris, Liang Pan, Xianfan Xu

**Affiliations:** 1 School of Mechanical Engineering and Birck Nanotechnology Center, Purdue University, West Lafayette, IN USA; 2 Charles D. Davidson School of Chemical Engineering, Purdue University, West Lafayette, IN USA; 3 Department of Chemistry, Purdue University, West Lafayette, IN USA

**Keywords:** Lithography, Laser material processing

## Abstract

There is demand for scaling up 3D printing throughput, especially for the multi-photon 3D printing process that provides sub-micrometer structuring capabilities required in diverse fields. In this work, high-speed projection multi-photon printing is combined with spatiotemporal focusing for fabrication of 3D structures in a rapid, layer-by-layer, and continuous manner. Spatiotemporal focusing confines printing to thin layers, thereby achieving print thicknesses on the micron and sub-micron scale. Through projection of dynamically varying patterns with no pause between patterns, a continuous fabrication process is established. A numerical model for computing spatiotemporal focusing and imaging is also presented which is verified by optical imaging and printing results. Complex 3D structures with smooth features are fabricated, with millimeter scale printing realized at a rate above 10^−3^ mm^3^ s^−1^. This method is further scalable, indicating its potential to make fabrications of 3D structures with micro/nanoscale features in a practical time scale a reality.

## Abstract (web summary)

Spatiotemporal focusing of ultrafast laser pulses enable thin print layers for projection multiphoton lithography. Complex 3D microstructures are printed in a continuous manner at high print rates.

## Graphical abstract

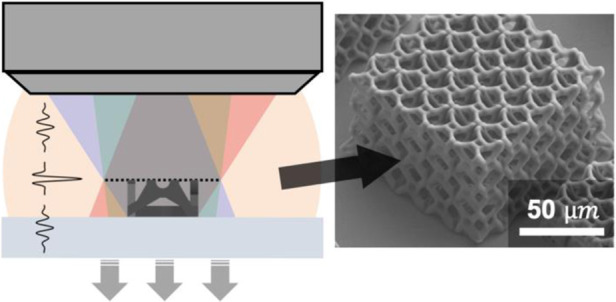

## Introduction

The technology of 3D printing has opened the door to a plethora of advances. One form of 3D printing, multi-photon lithography (MPL), has gained considerable popularity for the ability to arbitrarily structure 3D objects of micro/nano scale size with sub-micrometer resolution^1^. MPL has spurred advances in areas such as nanophotonics^2–5^, microoptics^6–10^, microrobotics^11–14^, mechanical metamaterials^15–18^, microfluidics^19–21^, and bioengineering^22–24^. The basic operation of this attractive printing process is the photopolymerization of a light sensitive resin via an ultrafast laser^25^. The polymerization is confined to the laser focus as a result of a nonlinear absorption of two or more photons, and the desired 3D structures are realized by scanning the laser focus through the resin. While useful for research and proof-of-concept applications, this method of scanning a single point does not lend itself well to scaling up printing throughput which is required for translating the many proposed microscale effects to the macroscale. Therefore, a significant challenge is presented in how to make this unique printing process more feasible for large scale manufacturing.

Several methods have been proposed for increasing the printing speed of the MPL process. One method to accomplish this is to increase the number of laser foci^21,26–34^. Recently, a 9-spot printing system was presented that utilizes an efficient 3D printed diffractive optical element which achieved a 10^7^ voxel s^−1^, or slightly larger than 10^−3^ mm^3^ s^−1^, printing rate^34^. This method and similar ones, however, are limited to fabricating periodic structures. In a different direction, many works implement holography to fabricate 2D patterns at once, reducing the translational requirements of the light for printing to one dimension, or sometimes none^35–39^. Similarly, direct image projection of 2D patterns has been demonstrated^40–43^. Whereas the method of 2D projection printing has proven valuable for scaling up single photon printing methods^44–46^, when applied to multi-photon processes the problem arises of how to confine the MPL to a thin plane as there will be undesired regions above and below the pattern plane with sufficiently large intensity to induce polymerization. Several works have attempted to address this problem by implementing a temporal focusing effect through the use of a grating in the optical system to create a strong laser pulse intensity gradient along the projection axis^40,41^. A recent work simplified the optics by using a digital micro-mirror device (DMD) and combined this with an efficient photoinitiator system to increase the fabrication speed^43^. Temporal focusing is achieved by separating the component wavelengths of a femtosecond laser pulse and then recombining them at a single plane. Under these conditions, the laser pulse becomes stretched in time when the component wavelengths are separated, reducing the pulse intensity. As the pulse approaches the pattern plane, the pulse becomes shorter until it reaches the focus where all the component wavelengths recombine to form the shortest, highest intensity pulse. After further propagation the wavelengths separate out again, reducing the intensity. Previous implementations of this technique were limited by the repetition rate of the laser used (1 kHz) as well as a layer-by-layer approach with laser off time between each layer.

In this work a continuous, layer-by-layer projection two-photon lithography system was constructed using a DMD to provide dynamic patterning as well as angular dispersion for temporal focusing of femtosecond laser pulses. This was done together with the development of a numerical model that accurately captures all relevant optical processes involved in the combined spatiotemporal focusing setup. With this numerical model, an estimated minimum layer thickness for large area 2D patterns was determined from the light intensity profile and compared to experiment. A 5 kHz laser repetition rate was utilized for faster patterning, leading to a continuous projection system without laser off time between layers. Fabrication of complex 3D structures with curved features and smooth surfaces were demonstrated. Scalability of the printing process was demonstrated with the fabrication of a complex metamaterial-like structure of millimeter scale.

## Results

### Continuous, layer-by-layer projection two-photon lithography system

**Fig. 1:**
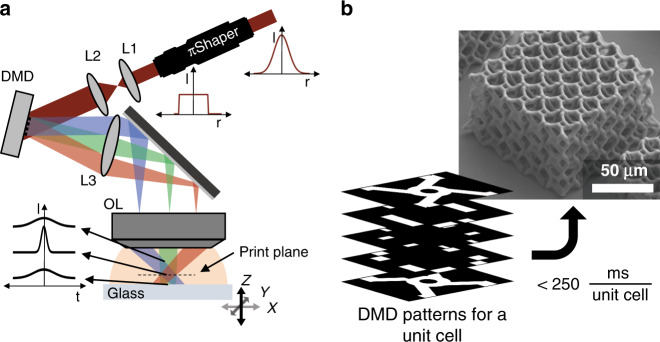
Continuous, layer-by-layer projection two-photon lithography system. **a** Schematic of spatiotemporal printing system, omitting mirrors and observational imaging system. A Gaussian spatial laser profile was transformed to a flattop intensity via πShaper. Lens pair L1 (*f* = 100 mm) and L2 (*f* = 150 mm) expanded the beam diameter to fill the DMD active area. Lens L3 (*f* = 300 mm) collected the diffracted light, and an objective lens (OL) reformed the DMD pattern at the print plane inside a liquid photoresist. A glass substrate was translated along 3 axes during fabrication. Laser pulse intensity was highest at the print plane and dramatically reduced both before and after the print plane as a result of temporal focusing. **b** Example of select DMD patterns displayed during 3D fabrication of a single 3D unit cell and the resulting 5 × 5 × 3 unit lattice structure

In our projection printing setup, temporal focusing was combined into a spatiotemporal focusing scheme through the use of a DMD as depicted in Fig. 1a. The DMD (DLP3000) is a dynamically patternable device, with patterning rates up to 4 kHz, providing the 2D patterns used for printing. Additionally, as the DMD operates as a grating to direct the light, it inherently adds the desired angular dispersion required for temporal focusing. The dispersion is represented by the separate-colored beams in the figure. For the printing, an 800 nm laser (Spectra-Physics Spitfire) with 22 nm bandwidth and 5 kHz repetition rate was used. A collecting lens (*f* = 300 mm) and microscope objective (100×, Nikon N.A. 1.49) were used to recreate the DMD pattern at the focal plane of the objective lens, referred to as the print plane. This spatial focusing is simultaneous with the temporal focusing which both occur at the print plane as a result of the pattern and dispersion originating from the same conjugate plane at the DMD. In order to provide uniform illumination of the DMD, a πShaper (AdlOptica πShaper 6_6_TiS) was used to transform the Gaussian spatial distribution of the femtosecond laser to a flattop profile which was then expanded to fill the entire DMD active area. The print plane was formed inside a photoresist with the objective lens oriented in a dip-in configuration to avoid limitations on ultimate fabrication size^47^. As MPL requires high peak laser intensities to operate, achieving polymerization across the large pattern area generated by the DMD implies that large laser powers are required. This would be challenging for the femtosecond oscillators commonly employed in MPL processes. Instead, a regeneratively amplified femtosecond laser is used which can achieve the peak power necessary for projection printing. Another limiting factor for achieving high printing rates is the sensitivity of the photoresist. Much work has been done to develop two-photon photoinitiators with improved photoinitiating capabilities^48–51^. Here, the recently introduced photoinitiator, (2E,6E)-2,6-Bis (4-(dibutylamino)benzylidene)-4-methylcyclohexanone (BBK), was used in the monomer, pentaerythritol triacrylate (PETA), at a loading of 0.7% (by weight)^49^. Structures are fabricated on a glass substrate which was translated in 3D space by high-speed, air-bearing precision stages (Aerotech ABL1000 series). Actual 3D printing was done by coordinating the stage motion with patterns being displayed on the DMD. A few select DMD images are shown in Fig. 1b for creating a metamaterial-like 3D unit cell. This cell was fabricated adjacently, multiple times, to create a metamaterial-like structure. A vertical stage speed of 100 µm s^−1^ was used to print each unit cell in less than 250 ms. Movie S1 (see Supporting Information) shows the fabrication of a similar structure under the same conditions. Faster speeds up to 1 mm s^−1^ have been used which will be shown later.

### Numerical model for spatiotemporal focusing

**Fig. 2:**
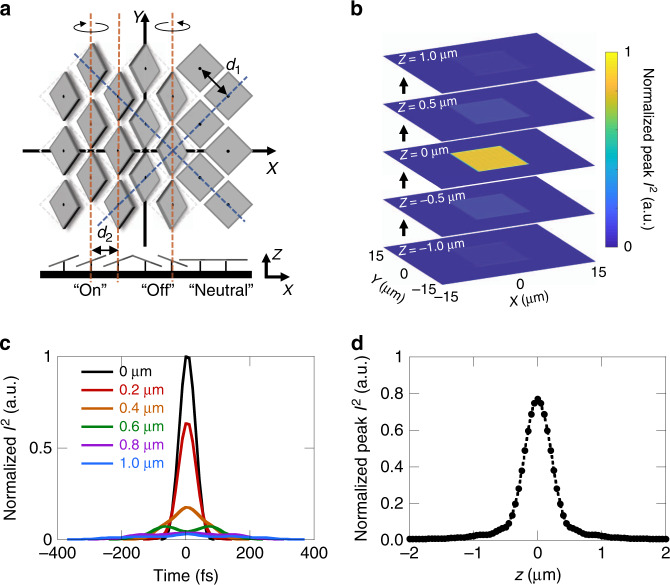
Simulation of spatiotemporal focusing. **a** DMD grating orientation. Blue dashed lines indicate the 2D grating with separation *d*1 formed by the mirror grid spacing. Orange dashed lines indicate the 1D grating with separation *d*_2_ formed by mirror tilt. Bottom part of figure indicates corresponding DMD mirror tilts for pixels in the “on,” “off,” and “neutral” states. **b** Simulated *xy*-plane profiles of peak *I*^2^ for progressive *z*-positions along the propagation direction of the spatiotemporal focusing near the print plane for a 1.52 mm × 2.28 mm rectangular pattern of DMD pixels being spatially and temporally focused. **c** Normalized *I*^2^ of the laser pulse profile in time for select *z*-positions along the center (optical axis) of the pattern from **b**. **d** *I*^2^ values for different *z*-positions along the center (optical axis) of the pattern from **b**

A numerical model was developed to evaluate the effectiveness of the spatiotemporal focusing in confining the patterned light sheet to a thin region for controlled print layer thickness. Monochromatic coherent Fourier optics with paraxial approximations were used to simulate the individual component wavelengths of the laser and then the results were combined to achieve the final light field. This was similar to previous modeling of the femtosecond projection two-photon lithography process^43^, however, several changes were introduced. The DMD can be considered as a 2D grating with period spacing *d*_1_ corresponding to the mirror spacing (blue dashed lines in Fig. 2a) and a diffraction order *m*_1_ for the propagating beam. However, as illustrated in the figure, the DMD pixels tilt along their diagonals during operation with the *x* and *y* axes oriented with the displayed pattern axes. The tilting mirrors form columns, indicated by the orange dashed lines, which act as a 1D grating with spacing *d*_2_. The spacing of this 1D grating is significantly smaller than the 2D grating spacing of the DMD pixels and contributes a stronger dispersive effect. Hence, the dispersion of the DMD was simulated in this work as a 1D grating in the form of an applied phase on the light field, $\phi _{DMD} = e^{2\pi jm_2\frac{{x_d}}{{d_2}}\frac{{\left( {\lambda - \lambda _0} \right)}}{\lambda }},$where *x*_*d*_ is the *x*-coordinate of the DMD, *m*_2_ is the diffraction order of the light propagation for the 1D grating, and *λ* is the wavelength component of the light with a corresponding center wavelength *λ*_0_. Supplementary Note 1 (see Supporting Information) provides a detailed description of the 1D grating model and shows that the calculated results obtained using the 1D grating effect agreed with the experimental observations (see Fig. S1, Supporting Information). Additionally, the incident angle of the laser on the DMD to achieve a blazed diffraction order propagating along the DMD surface normal resulted in a pulse front tilt (PFT) with a 3.65 fs µm^−1^ delay across the pulse area (see Fig. S2, Supporting Information). This PFT effect could be visualized as each pulse performing a fast line scanning of the sample plane^52^. In order to account for this, a corresponding phase delay, $\phi _{{\mathrm{PFT}}} = e^{ - 2\pi j\frac{{D_{{\mathrm{delay}}}}}{\lambda }},$ was also applied to each wavelength across the columns of the DMD with the additional distance traveled by the light for each column represented by *D*_delay_. Details are provided in Supplementary Note 1 (see Supporting Information).

To gain insight into the light field confinement for continuous, layer-by-layer projection two-photon lithography, a large area rectangular pattern on the DMD of 1.52 mm × 2.28 mm was simulated. The resulting peak light field intensity square, *I*^2^, is shown in Fig. 2b at the print plane as well as at several planes above and below the print plane. Intensity square was used to better represent the strength of the two-photon absorption that must occur for printing. The peak value was mapped instead of the time averaged intensity as the average intensity would not accurately capture the threshold effect of absorption due to the multi-photon absorption during each individual pulse in time. All values were normalized to the maximum value found at the print plane. The temporal focusing effect was clearly captured by the simulation as shown in Fig. 2c, where the time domain pulse profile was examined at several distances from the *z* = 0 print plane for a location at the center of the propagating pattern. The intensity falls off rapidly up to 0.6 µm from the print plane and, more demonstrative of the temporal focusing, the pulse profile shows clear broadening of the width. The broadening continues further from the print plane (see Fig. S3, Supporting Information). The peak *I*^2^ values along the optical axis in the center of the pattern are plotted in Fig. 2d. The field drops off rapidly within a 1 µm range around the print plane. With the light field confined well within such a range, the ability to print thin 2D planes on the scale of single micrometer thickness could be reasonably expected.

### Analysis of spatiotemporal printing features

**Fig. 3:**
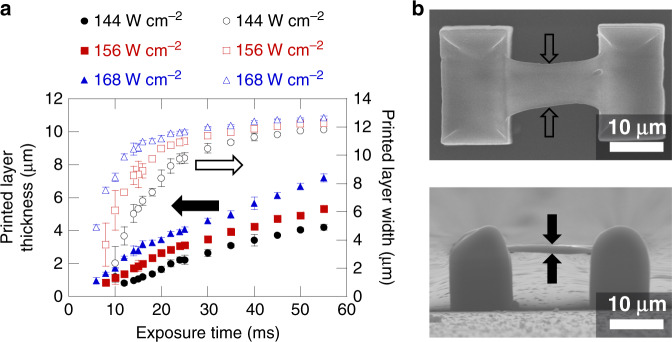
Printing of single layers. **a** Printed layer thickness and width for varying exposure times and laser intensities. DMD pattern of 2.28 mm width was used for printing all layers. Each layer was printed suspended across two prefabricated supports. Error bars indicate one standard deviation of measurements. **b** Example scanning electron microscope (SEM) images of printed layer structure using 156 W cm^−2^ intensity and 14 ms exposure viewed from top (upper image) and side (lower image)

For comparison with the simulated pattern, a large rectangular pattern spanning the length of the DMD area with width of 2.28 mm was chosen for printing single layers. Each layer was printed suspended across two prefabricated supporting structures. The exposure time for each layer was varied to determine the threshold of printing a quality pattern. This was repeated for several laser intensities. The laser intensities presented are the intensities estimated at the print plane. Both the width and thickness of the printed layers were measured, and the results are presented in Fig. 3a. For the demagnification of the printing system (150×) a printed rectangle width of 15.23 µm would be expected. The width of the patterns was measured at their center, as indicated in the upper part of Fig. 3b. Under increasing exposure time, the pattern width approached a constant close to 12 µm. This value does not change significantly as laser intensity was increased. A constant width is expected as the projected image has very sharp edges and there is minimal light exposure in the lateral direction outside the image region. The smaller than expected width was likely due to shrinkage of the prints after the development process, particularly evident in a pronounced “necking” effect that was observed at low exposure. The shrinkage is not unexpected for the PETA-based resist and can be mostly avoided by using low-shrinkage hybrid resists such as SZ2080^53^. Unfortunately, BBK was found not to mix well with SZ2080 for the purpose of increasing efficiency and additionally SZ2080 is not suitable for dip-in lithography. As exposure time was reduced, the width reached a point at which it started significantly reducing, indicating a threshold region of exposure for obtaining a print representative of the target DMD pattern. The print thickness was measured as shown in the lower part of Fig. 3b. A print thickness of just over 2 µm was observed at the point which the pattern begins to significantly deviate from the target shape. For longer exposure times, the print thickness continues to increase though at a slightly reduced rate. The thickness is ultimately expected to plateau due to the lack of sufficient laser intensity further away from the print plane resulting from the spatiotemporal focusing. Before that point, the thickness continues to grow because the local oxygen is being depleted in a large volume around the print while the laser is on, reducing the threshold intensity required for polymerization. Fig. S4 (see Supporting Information) shows a representative set of images for the 156 Wcm^−2^ laser intensity, with significant visible change occurring in the pattern for exposures below 20 ms. It is likely that reducing the shrinkage properties of the resist will allow thinner individual layers approaching the thickness suggested by the simulation to be printed while maintaining the pattern shape. Already, thicknesses less than 1 µm were achieved here for non-ideal pattern shapes, indicating a micron-scale or less axial feature size for 2D planar prints. This is separate from the axial resolution for large 2D layers, which is the minimum separation between two layers while maintaining a complete gap. An investigation of the axial layer resolution (see Fig. S5, Supporting Information) showed a slightly larger value than the feature size which is expected due to the proximity effect observed in two-photon lithography^54^.

**Fig. 4:**
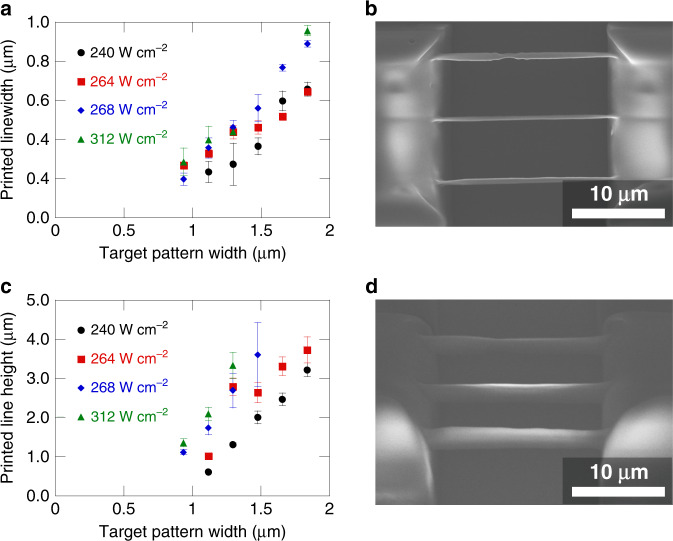
Printing of suspended line structures. **a** Printed linewidths as a function of projected pattern width at the print plane. Each point represents an average of 3 lines printed suspended across preprinted supports. Error bars represent one standard deviation of measurements. **b** Example printed structure from **a** for target 1.296 µm width lines printed with 264 W cm^−2^. **c** Printed line heights as a function of projected pattern width at the print plane. Each point represents an average of 3 lines printed suspended across preprinted supports. View angle is at 45˚ from substrate normal. Error bars represent one standard deviation of measurements. **d** Example printed structure from **c** for target 1.116 µm width lines printed with 264 W cm^−2^. All line prints were performed with a stage travel speed of 100 µm s^−1^ and a DMD pattern exposure of 5 ms

An advantage of MPL is the ability to fabricate structures with submicron features. To test this ability with the projection two-photon lithography process, patterns of thin suspended lines were fabricated. The width of the fabricated lines is plotted against the target width of the DMD pattern in Fig. 4a with an example set of lines shown in Fig. 4b. The actual printed width was significantly smaller than the target width and this trend holds true even for larger target linewidths (see Fig. S6a, Supporting Information). This is attributed to the combination of strong inhibition processes at the edge of the pattern and structure shrinkage^54–56^. Nonetheless, linewidths of less than 200 nm were achieved, demonstrating the submicron featuring capability of the process on the same order as traditional multiphoton printing processes. The linear trend of increasing linewidth with projected pattern width is expected as the line lateral dimensions should be primarily defined by the pattern dimensions since this is an image projection process. Additionally, the height of the printed lines was measured in Fig. 4c, demonstrating a minimum line height less than 1 µm. This is comparable to line heights achieved with traditional multiphoton lithography^57^. An angled view in Fig. 4d shows an example view of the line heights. The line heights rapidly increased as the pattern width increased. However, the height started to plateau between target widths of 2 to 3 µm (see Fig. S6b, Supporting Information). A possible reason is that at small pattern widths the inhibition effects in the photoresist dominate due to diffusing oxygen and thus the spatiotemporal focusing is not as prominent in the resulting structure. However, once a critical area of exposure is reached, inhibition can no longer confine the polymerization due to depletion of oxygen in the center of the area. At this point the spatiotemporal focusing becomes the dominant limiting effect and clearly confines the printing to a finite plane regardless of further increasing pattern area. This inhibition effect has been attributed to lower laser power requirements for printing larger 2D areas previously^41,43^.

### Continuous, layer-by-layer fabrication of complex 3D structures

**Fig. 5:**
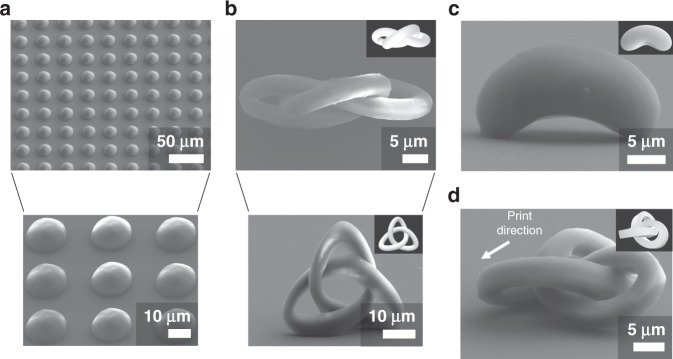
3D structures printed using continuous, layer-by-layer projection two-photon lithography system. All structures were fabricated with a 100 µm s^−1^ stage speed along the vertical (*z*) direction. Computer models used for printing are included as figure insets for complex structures. **a** Curved surfaces demonstrated through fabrication of array of hemispheres comparable to microlens arrays. 1 mm^2^ area of coverage in about 6.2 min with 1 mm s^−1^ stage travel between each hemisphere and laser intensity of 132 W cm^−2^. **b** Complex trefoil knot structure with a circular cross-section viewed from top (upper image) and 75˚ tilt angle from normal (lower image) printed with 144 W cm^−2^ laser intensity. **c** Chicago’s Cloud Gate sculpture printed with 120 W cm^−2^ laser intensity demonstrating largescale curved features. **d** Trefoil knot with square cross-section printed with 144 W cm^−2^ laser intensity. The structure fell over during the development process, therefore the print direction is indicated

The planar printing capabilities of the projection printing setup discussed above enabled continuous, layer-by-layer 3D printing. The DMD was used to generate multiple patterns in sequence from 2D slices of a CAD profile resulting in the formation of 3D printed structures. The DMD pattern slices were chosen with separations of 125–420 nm, much smaller than the ~1 µm estimated minimum layer thickness. This ensured a smooth print surface with no layering artifacts on the sides. The individual DMD mirrors translated to ~51 nm size at the print plane, which is below the diffraction limit of the system allowing smooth, curved patterns to be printed without being limited by the DMD resolution. For example, circular patterns of decreasing radius were printed sequentially to form hemispheres representative of microlenses, as in Fig. 5a. Using a vertical stage speed of 100 µm s^−1^ a single hemisphere was fabricated in less than 120 ms. Uniform arrays of hemispheres were fabricated this way, covering a 1 mm^2^ area in 6.2 min, including the time for lateral stage motion between each structure. No layering effects were found in the surfaces of the hemispheres. Some small roughness observed was due to laser speckle in the spatial beam profile as a result of some dirty or damaged optics. Hemispheres of varying diameter can be printed simply by changing the DMD patterns. More complex, curved features can also be printed as demonstrated by the fabrication of the trefoil knot structure in Fig. 5b. The lateral features matched the target structure well in the top-down view showing the high resolution of the projection two-photon lithography process in the *xy*-plane. A side view of the trefoil knot showed a good matching with the target profile, with some elongation as a result of the larger minimum achievable features in the *z* direction. The ability to print aspherical geometries was demonstrated in Fig. 5c by the nanoscale Cloud Gate sculpture. Figure 5d shows again the fabrication of a trefoil knot, this time with a square cross-section illustrating the ability to print sharper features. The real-time fabrication of a trefoil knot can be observed in Movie S2 which shows that the trefoil knot was made within 319 ms, and the same video with 10× reduced playback speed in Movie S3 (see Supporting Information). All of the structures in Fig. 5 were printed using 100 µm s^−1^ stage speed. Similar print quality can be achieved with more well-known photoinitiator 7-diethylamino-3-thenoylcoumarin (DETC) at the cost of significantly higher laser intensities (see Fig. S7, Supporting Information).

### Scalability and large volume printing

**Fig. 6:**
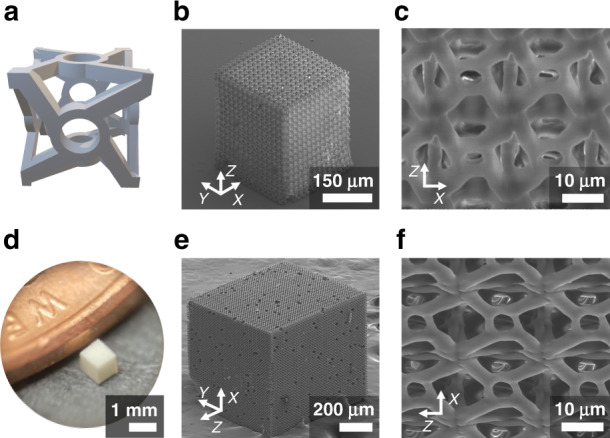
Rapid 3D printing of macroscale metamaterial-like structures. **a** 3D CAD profile of material unit cell. **b** 15 × 15 × 15 unit metamaterial-like structure fabricated with 400 µm s^−1^ vertical stage speed for each unit. **c** Magnified view of side of structure in **b** with view angle about 57° from substrate normal. **d** Optical image of 42 × 42 × 42 unit metamaterial-like structure situated near the edge of a United States one-cent coin. Each unit cell was fabricated with 1 mm s^−1^ vertical stage speed. Including all stage motions at the same 1 mm s^−1^ speed, total print time was about 2.3 hours. **e** SEM image of structure from **d**. **f** Magnified view of side of structure in **e** with view angle about 65˚ from substrate normal. Holes in structures are missing unit cells where DMD failed to trigger and display patterns during printing

Scalability of the printing process was evaluated through the fabrication of a metamaterial-like structure. The unit structure chosen for this Fig. (6a) was similar in design to a recently introduced chiral metamaterial that has been chosen by others to demonstrate the speed of a multiphoton printing process^16,34^. Only a visual “likeness” to that chiral metamaterial is suggested for the structure here, as no evaluation of the structural properties were performed in this work. This structure features straight and curved components in all 3 dimensions as well as close separations along the *z* direction making it an ideal choice for demonstrating the capabilities of the print process. A vertical stage speed of 400 µm s^−1^ for printing and a 1 mm s^−1^ stage translation speed between units were used to fabricate the 15 × 15 × 15 unit metamaterial-like structure in Fig. 6b with laser intensity 216 W cm^−2^. A close-up image of the side is displayed in Fig. 6c with the unit cell structure clearly visible. For comparison, a 42 × 42 × 42 unit metamaterial-like cube structure corresponding to a 1 mm^3^ volume was fabricated with an increased print speed of 1 mm s^−1^ and laser intensity of 312 W cm^−2^ in 2.3 hours total print time, with the lateral stage motion (motion between printing each unit cell) accounting for almost 80% of total time. Figure 6d is an optical image of the printed cube next to a United States penny. An SEM image of the cube is shown in Fig. 6e. The cube is on a side in the image with the print direction having been along the indicated *z*-axis. Again, under closer visual inspection of the side of the structure in Fig. 6f, the printed unit cell is clearly visible with comparable quality to the 400 µm s^−1^ printed structure in Fig. 6c. This demonstrated that printing with continuous, layer-by-layer projection two-photon lithography can be scaled up without significant changes to the resulting structure quality. The quality of millimeter scale prints was shown to be consistent throughout the entire printed volume by examination of the interior via laser cutting (see Fig. S8, Supporting Information).

With the demonstrated printing stage speed of 1 mm s^−1^ and an available print area of 43.81 µm × 24.66 µm (determined by the active DMD area and system optics) the projection two-photon lithography system introduced here achieved a 1.08 × 10^−3^ mm^3^ s^−1^ volumetric printing rate (see Supplementary Note 5). This rate is ~5× faster than the previously reported speed using a similar method^43^ when accounting for the differences in available projection area. The increase in speed arises from the removal of the step time between printing individual layers, which previously was longer than the time of pattern exposure, instead continuously translating the stage while changing 2D patterns. Additionally, the voxel print rate for this system was 3.2 × 10^6^ voxels s^−1^, determined by printing a woodpile structure at the same 1 mm s^−1^ stage speed (Fig. S9). A 16 × 16 pixel unit on the DMD corresponded to a printed voxel as a result of the 16 pixel wide patterns in the woodpile. The fabricated woodpile has a 380 nm lateral voxel dimension which means the volumetric print rate and the voxel print rate are both determined for submicron feature structuring. This voxel print rate is already greater than the 3.2 × 10^4^ voxel s^−1^ rate^58^, and approaches the 10^7^ voxel s^−1^ rate^34^, achieved by previous works. Moreover, the volumetric print rate can be easily increased further by larger DMD area and lower magnification optics. The process shows no clear limit to the scalability under the conditions tested here, however DMD pattern rate and active area, as well as available laser power, are important factors that will ultimately create a bottleneck.

### Structuring potential of projection two-photon lithography

**Fig. 7:**
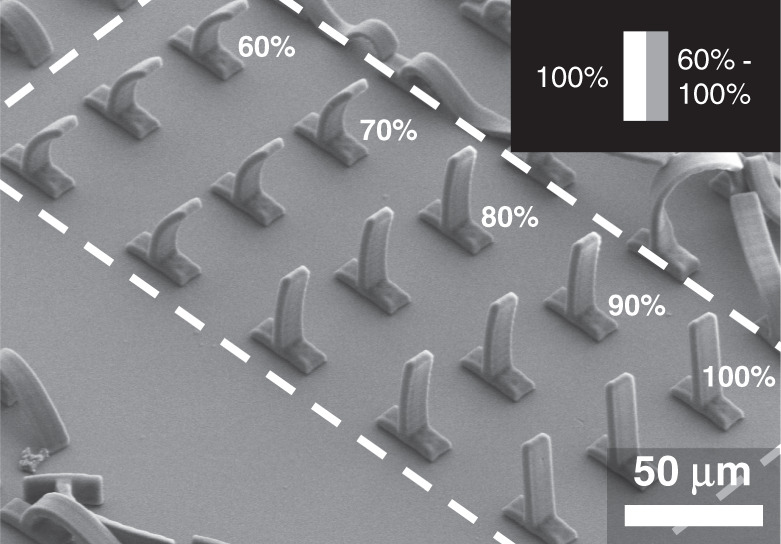
Tuning structure characteristics using grayscale projection. Grayscale pattern projection demonstrating tunable material properties in printing of standing pillars. Percentages indicate grayscale level of the pattern shown in the inset. Print speed was 500 µm s^−1^ stage speed

The DMD is capable of not only printing binary patterns but can also introduce grayscale into the patterning. This is a desirable feature for tuning the exposure dose across the part to be printed, which has previously been demonstrated by varying the laser power during printing^59^ and allows the fabrication of metamaterial structures with unique properties^17^. Grayscale patterning using the projection printing system here is presented in Fig. 7b. Micropillars are fabricated using the DMD pattern shown in the figure inset. Half of the pillar is fully exposed at 100% grayscale value and the other half is varied from 60% to 100%. For lower grayscale values, there is less crosslinking of the photoresist which leads to a larger amount of shrinkage after development. With this pattern, the result is a curvature of the pillars due to residual stresses created by the nonuniform shrinkage in the structure. The amount of curvature is tunable by controlling the grayscale value as demonstrated in the figure. These pillars were fabricated using 500 µm s^−1^ stage travel speed. The grayscale effect does require slower print speeds to allow for the change in exposure dosage, however the demonstration here shows relatively high print speeds can still be achieved. Additionally, going beyond the relatively simple, repeating structures shown in Fig. 6, the continuous, layer-by-layer projection two-photon lithography is also capable of fabricating large-scale aperiodic structures. This is demonstrated in Fig. S10 which shows a structure composed of two unit cells of different sizes. By changing unit cell sizes, the structure outlines the ability for fabrication of gradient density structures. Important to note is that the unit cells in this structure are larger than the projection area of the DMD in the system. Thus, they are fabricated in multiple unique pieces, showing that the system is not limited to printing periodic structures. Each part is stitched together by a partial overlap with the neighboring volumes, the process of which is outlined in Supplementary Note 6.

## Discussion

We showed a continuous, layer-by-layer projection two-photon lithography printing system for high-speed fabrication of polymer 3D structures with micron and submicron scale features. A numerical model was developed that captured all relevant effects for determining the light field intensity confinement for a large area planar projection including dispersion effects from the DMD and the pulsefront tilt imparted on the laser pulse. Utilizing the high-performance BBK photoinitiator chemistry, the print layer thickness and resolution for the printing system yielded good correlation with simulation. Furthermore, complex 3D geometries including smooth, curved surfaces were fabricated in a continuous fashion on the millisecond timescale. The rapid 3D printing of a metamaterial-like structure of millimeter scale was achieved, demonstrating a print speed of 1.08 × 10^−3^ mm^3^ s^−1^ which is comparable to current state-of-the-art MPL processes, but is capable of printing arbitrary 3D structures. The print area, and therefore print speed, can be increased further by simple changes in the system optics, indicating that this printing process is a promising direction for the scaling up of 3D nanoprinting throughput.

## Materials and methods

### Materials

Chemicals including methanol, 4-(Dibutylamino)benzaldehyde, 4-methylcyclohexanone, potassium hydroxide, and PETA were purchased from Sigma–Aldrich. DETC was purchased from J&K Scientific. All above chemicals were used as received.

### Synthesis and preparation of photoresists

The photoinitiator (2E,6E)-2,6-Bis (4-(dibutylamino)benzylidene)-4-methylcyclohexanone (BBK) was synthesized via an aldol condensation reaction, as reported previously^49^, and characterized by ^1^H-NMR (see Fig. S11, Supporting Information) and ultraviolet–visible (UV–Vis) light spectroscopy (see Fig. S12, Supporting Information). Photoresist mixtures were created by mixing a photoinitiator with the monomer PETA and sonicating overnight.

### 3D printing system

A 65 fs regeneratively amplified laser (Spectra-Physics Spitfire) with 5 kHz repetition rate, 800 nm center wavelength, and ~22 nm bandwidth was used as the printing laser. A half-waveplate (Newport) and polarizing beam splitter cube (Thorlabs PBS25-780) provided power modulation. The beam was expanded to ~6 mm diameter using a planoconcave (*f* = −75 mm, Thorlabs LA1582-B) and planoconvex (*f* = 100 mm, Thorlabs LA1509-B) lens pair. The beam was passed through a πShaper (AdlOptica πShaper 6_6_TiS) and then expanded using two planoconvex lenses (*f* = 100 mm and 150 mm, Thorlabs LA1509-B and LA1433-B) before being incident on the DMD (DLP3000) at ~24° from DMD surface normal. Laser pulse duration was minimized before the DMD surface using a GaP photodetector (Thorlabs DET25K) and adjusting the amplifier’s internal grating compressor. Diffracted light along the DMD surface normal was collected by an achromatic doublet (*f* = 300 mm, Thorlabs AC254-300-B-ML) and passed to the microscope objective lens (Nikon 100×, N.A. = 1.49). A 50/50 beam splitter (Thorlabs BSW29) was placed before the objective lens for in situ imaging with a charge coupled device (CCD, Panasonic). The objective lens was dipped into the photoresist mixtures which were drop cast on a microscope slide substrate. The substrate was positioned using a 3-axis air bearing stage (Aerotech ABL1000 series). For 3D printing, a set of patterns were uploaded to the DMD which was then externally triggered by the stage motion control software to synchronize the display and motion. The substrate surface was located for printing using a 633 nm laser introduced to the beam path via dichroic beam splitter (Thorlabs DMLP650). The provided laser intensities were the estimated intensities at the print plane. The intensity values were determined by measuring the laser power entering the back of the objective lens using a “white” screen on the DMD in which all the pixels were in the ‘on’ state and assuming 70% transmission through the objective lens.

### Post-print processing

After printing, samples were placed in an isopropanol (IPA) bath for ~15 min before being transferred to a fresh IPA bath for another 5–10 min under a 465 nm LED source (Thorlabs M470L3) with intensity approximately 0.065 W cm^−2^. The exposure was intended to improve crosslink density of the printed structure to assist with surviving the development process. The sample was then allowed to dry in air. For the larger structures in Fig. 6 the IPA treatments were extended to several hours to ensure the complete removal of all unpolymerized resist. For only the 1 mm^3^ sized structure a critical point dryer (Tousimis Automegasamdri 915B) was used to dry the sample^55^. Samples were sputter coated with a Au/Pd mixture before being imaged with an SEM (Hitachi S-4800). Typical parameters for imaging were 15 kV and 5 mA.

## Supplementary information

Supplementary Information

Movie 1

Movie 2

Movie 3

## Data Availability

The data that support the results within this paper and other findings of the study are available from the corresponding authors upon reasonable request.
