# Supplementary material for: Rapid, continuous projection multi-photon 3D printing enabled by spatiotemporal focusing of femtosecond pulses
Source: Light Sci Appl. 2021 Sep 24;10:199. doi: 10.1038/s41377-021-00645-z (PMC8463698; doi:10.1038/s41377-021-00645-z)
Supplement: Supplementary file 1 — Supplementary Information [file 41377_2021_645_MOESM1_ESM.docx]

**Supplementary Information for Rapid, Continuous Projection Multi-photon 3D Printing Enabled by Spatiotemporal Focusing of Femtosecond Pulses**

Paul Somers,^1^ Zihao Liang,^2^ Jason E. Johnson,^1^ Bryan W. Boudouris,^2,3^ Liang Pan,^1^ Xianfan Xu^1^*

^1^School of Mechanical Engineering and Birck Nanotechnology Center, Purdue University, 1205 W. State St., West Lafayette, Indiana 47907, USA

^2^Charles D. Davidson School of Chemical Engineering, Purdue University, 480 W Stadium Ave, West Lafayette, Indiana 47907, USA

^3^Department of Chemistry, Purdue University, 480 W Stadium Ave, West Lafayette, Indiana 47907, USA

*Corresponding author: Xianfan Xu (xxu@ecn.purdue.edu)

Additional Supplementary Material

Movie S1. Movie of printing metamaterial-like structure with 3 × 3 × 3 units. Some ghost images of the structure being fabricated are visible from multiple surfaces of the beam splitter used for imaging.

Movie S2. Movie of printing square trefoil knot structure. Fluorescence of photoresist and scatter from laser are visible during fabrication. Stage returns to initial position after printing.

Movie S3. Movie of printing square trefoil knot structure at 90% reduced speed.

Supplementary Note 1: Numerical Model of Spatiotemporal Projection and Results

The optical setup of the digital micromirror device (DMD), collector lens, and microscope objective were modeled in a 4f-like configuration in which the DMD is at the focus of the collecting lens and the print plane is formed at the focus of the objective lens (modeled as a thin lens). The incident light field on the DMD was modeled as a plane wave composed of frequencies ω with a Gaussian distribution, $U_{\text{i}}\left(x_{\text{d}},y_{\text{d}},\omega\right)=Ae^{\frac{\left(\omega-\omega_{0}\right)^{2}}{\Omega^{2}}}$. Coordinates $x_{\text{d}}$ and $y_{\text{d}}$ are the DMD spatial coordinates, $\omega_{0}$ is the laser center frequency, *A* is the amplitude of the profile, and $\Omega=\frac{\Delta\omega}{2\sqrt{\ln2}}$, where ∆ω is the Full Width Half Max (FWHM) of the pulse spectrum. The DMD then imparts both an intensity distribution (the pattern) and an angular dispersion to the light field. The desired print pattern is represented as a binary mask function, $H\left(x_{\text{d}},y_{\text{d}}\right)$. The angular dispersion is represented as an applied phase term. The DMD could be considered as a 2D grating due to its rectilinear patterning of micromirrors which would suggest a phase term for the dispersion of , where the frequencies of the light field are converted to wavelength, $\lambda$, with a corresponding center wavelength $\lambda_{0}$, *j* is $\sqrt{-1}$, the diffraction order is represented by $m_{1}$, and $d_{1}$ is the period of the 2D grating. The cosine and sine terms rotate the dispersion direction to match the coordinate system. However, the main dispersion contributed by the DMD results from the tilting mirrors that form columns, indicated by the orange dashed lines in Fig. 2a, which act as a 1D grating with spacing $d_{2}$. The phase term used to represent the DMD dispersion is then given by Equation 1.

(1)

The 1D grating effect corresponds to a diffraction order, $m_{2}$, of the diffracted light field. The pulse front tilt (PFT) that arises from diffracting off the DMD is included with a phase delay that is applied to each wavelength across the columns of the DMD. The total extra distance the light field has to travel for each column along the face of the DMD is represented by $D_{\text{delay}}$. The PFT phase term is then given by Equation 2.

(2)

The light field leaving the DMD is then represented by . The field at the back entrance of the objective lens, $U_{\text{B}}$, is found by taking the Fourier transform of the field from the DMD, simulating propagation through the collecting lens of focal length $f_{1}$.

()

are the spatial coordinates at the back of the objective lens. After applying a pupil function, *P*, representing the back aperture of the objective lens, the final light field after the objective lens a distance *z* from the print plane is given by Equation 4.

(4)

Here *n* is the refractive index of the photoresist and $f_{2}$ is the focal length of the objective lens. $\left(x_{\text{f}},y_{\text{f}}\right)$ are the spatial coordinates at the print plane. Finally, the pulse intensity in the time domain is determined after taking a Fourier transform of the frequency components, .

A grid of 600 × 600 with pixel spacing $d_{1}$was used for the model. Final results were normalized to the maximum field intensity at the print plane. All parameters were experimental conditions (Table S1). Simulations were run using DMD phase terms for both the 2D grating and 1D grating effect as well as for each case individually. The light intensity was plotted at *z* = -6 µm for all cases and compared to the observed laser intensity profile from the CCD in the experimental setup (Fig. S1). Simulations of the 1D grating effect matched experimental observations extremely well, while simulations using the 2D grating effect deviated significantly. As a result, only the 1D grating term was included for all simulation results. The PFT accounted for in the simulation is depicted in Fig. S2, where the DMD is oriented in a blazed grating configuration. The simulated stretching of the laser pulse along the optical axis is presented in Fig. S3, which shows the full-width at half-maximum (FWHM) of the laser pulse profiles.

**Table S1 Numerical model parameters.** Relevant parameters matching experimental conditions used in the numerical model.

| Pixel Spacing of DMD | $d_{1}$ | 7.6 µm |
| --- | --- | --- |
| Column Spacing of DMD | $d_{2}$ | 5.4 µm |
| Center Wavelength | $\lambda_{0}$ | 800 nm |
| Diffraction Order of 2D Grating | $m_{1}$ | 4 |
| Diffraction Order of 1D Grating | $m_{2}$ | 3 |
| Spectral Bandwidth | $\Delta\lambda$ | 22 nm |
| Refractive index of Photoresist | *n* | 1.48 |
| Focal Length of Collecting Lens | $f_{1}$ | 300 mm |
| Focal Length of Objective Lens | $f_{2}$ | 2 mm |
| Pulse Front Tilt |  | 3.65 fs µm^-1^ |


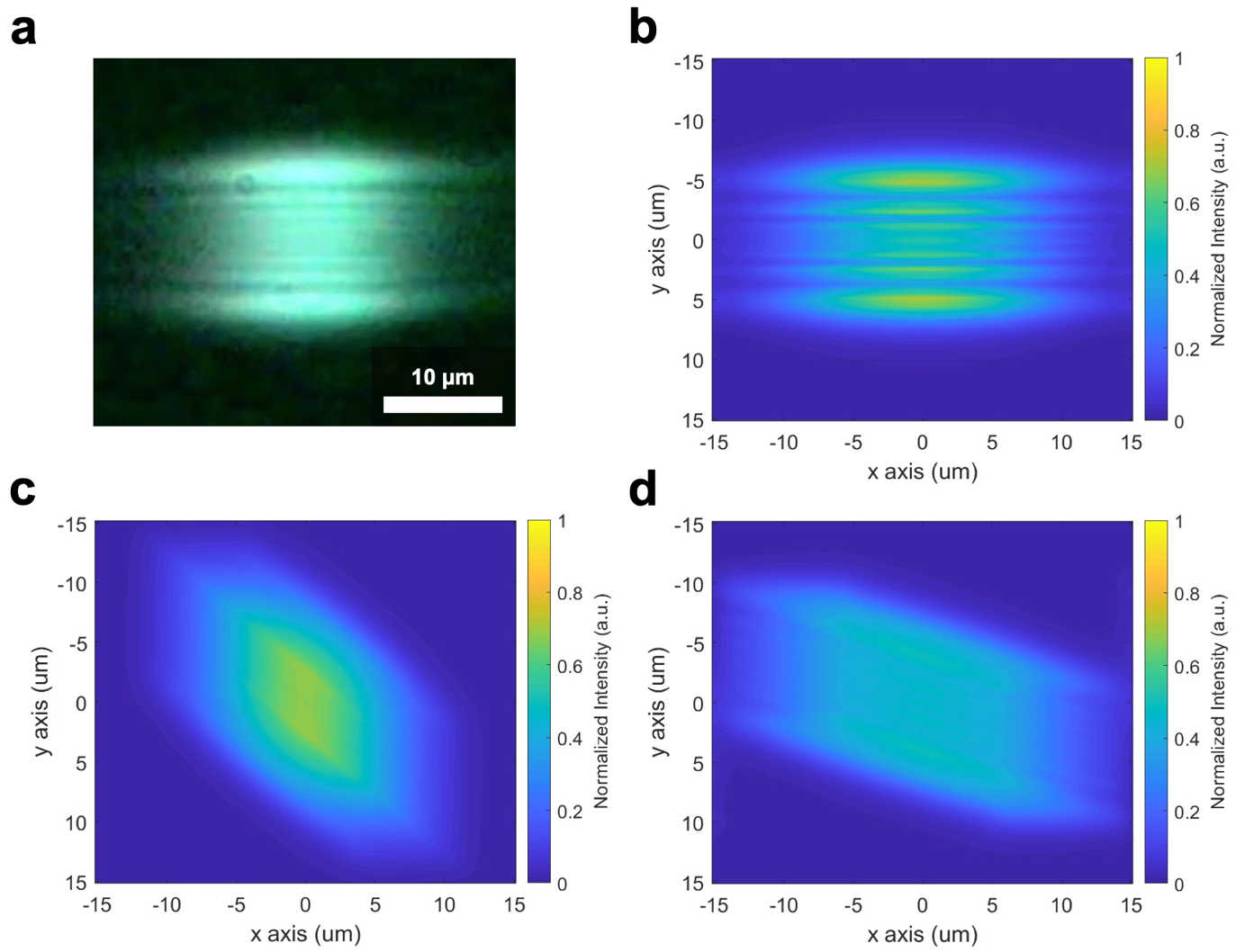


**Fig. S1 Comparison of numerical model to experiment.** Time averaged intensity profile of a 1.52 mm × 2.28 mm rectangular pattern of DMD pixels observed at *z* = -6 µm from the print plane. **a** Profile observed captured using CCD in experimental setup. **b** Simulated profile using only 1D grating phase term. **c** Simulated profile using only 2D grating phase term. **d** Simulated profile using both 2D and 1D grating phase terms.


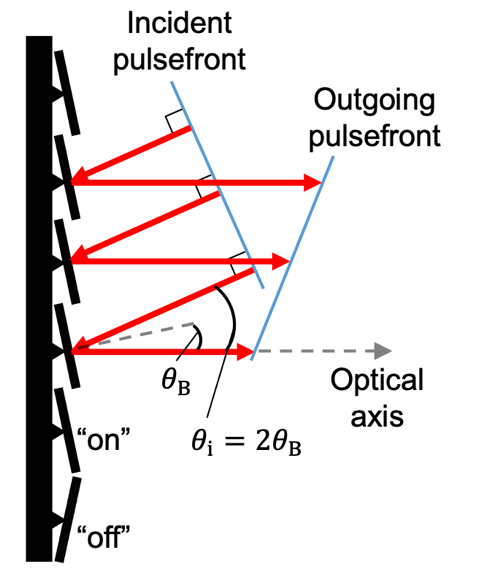


**Fig. S2 DMD induced PFT.** Schematic of the laser pulse front incident and diffracted from the DMD surface outlining the PFT picked up by the laser pulse. Blaze angle ($\theta_{\text{B}}$= 12°) of the DMD grating and laser incident angle ($\theta_{\text{i}}$) are indicated.


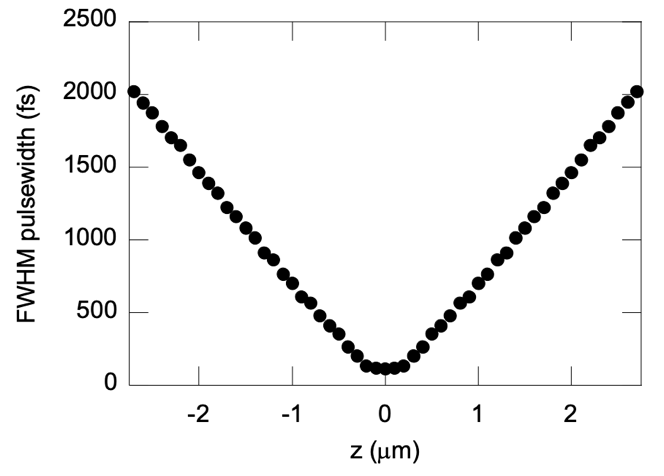


**Fig. S3 Pulsewidth change due to temporal focusing.** Full-width half-maximum (FWHM) pulsewidths taken along the optical axis for 1.52 mm × 2.28 mm rectangular pattern of DMD pixels being spatially and temporally focused for varying distances *z* from the print plane (*z* = 0).

Supplementary Note 2: Supplementary Projection Printing Results

Investigations into the print performance of the projection printing process was done by evaluating the performance of printing isolated features. An exemplary set of data for determining the ability to fabricate a thin single layer is presented in Fig. S4. The printed structure begins to lose its well-defined shape for laser exposures below about 20 ms.

In order to determine the resolution for printing individual layers, two layers were printed with varying separation between them (Fig. S5). Both patterns were printed during one motion of the stage at 100 µm s^-1^ and 5 ms of exposure for each pattern. The observed connecting polymer structure between layers with smaller separation is attributed to a double exposure of the region within a sufficiently short time such that the overall concentration of photoradicals produced overcome the threshold concentration required for polymerization. Under a single exposure that same region is below the required photoradical concentration to polymerize. It is likely that the addition of a stronger inhibiting process would reduce the radical concentration build up and allow finer layer separation.


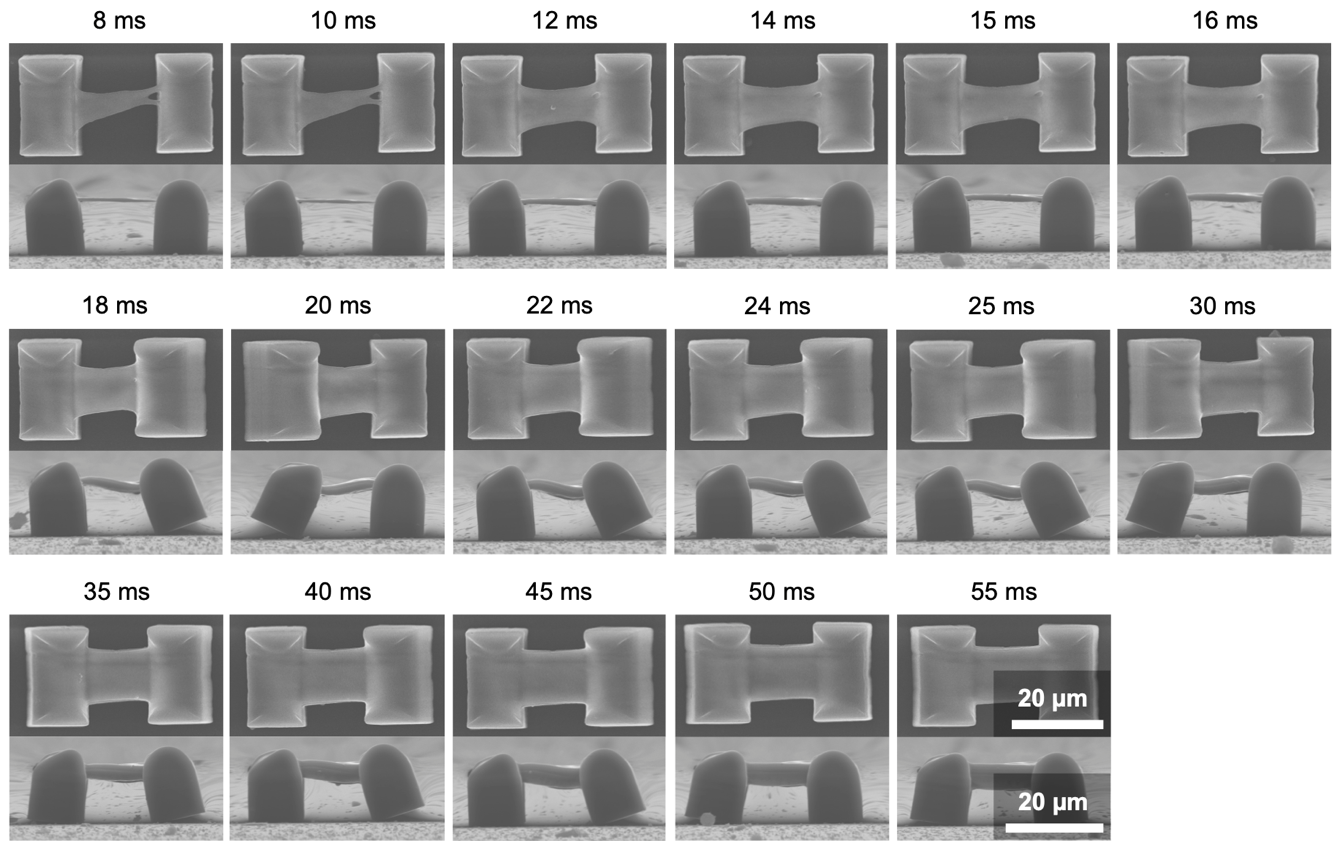


**Fig. S4 Single layer printing under varying print conditions.** Example data set from Fig. 3a for laser intensity of 156 W cm^-2^. The number above each image set is the corresponding pattern exposure time. Upper images are the view from top. Lower images are the view from side. Scalebars for both upper and lower images are 20 µm.


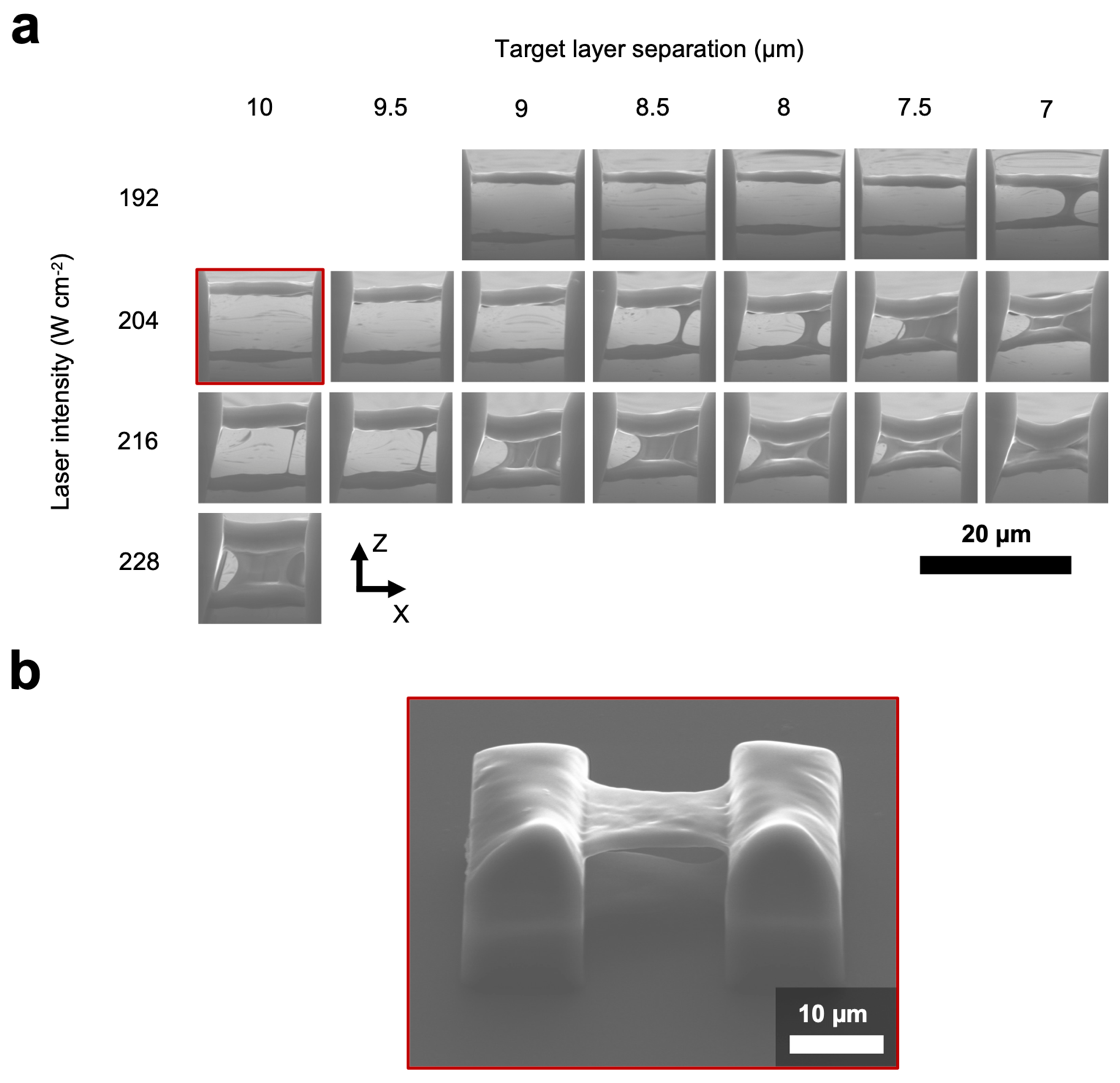


**Fig. S5 Determination of multi-layer printing resolution.** **a** SEM images of two printed layers separated by varying distance. Layers were fabricated suspended across prefabricated supports. A stage travel speed of 100 µm s^-1^ with a pattern exposure time of 5 ms for each layer was used. Scalebar is 20 µm. **b** View of red outlined structure from (**a**) at a 45˚ tilt angle from substrate normal. Scalebar is 10 µm.

The performance of the spatiotemporal focusing was evaluated over a large range of pattern widths and laser powers (Fig. S6). The width and height of printed line structures was measured. All measured widths were below the target size, however patterns printed with larger laser intensities began to approach a slope closer to the target slope. Measured line heights all begin to plateau within a target pattern width of 2-3 µm. Oxygen inhibition is likely a dominating confining process below this width, whereas the spatiotemporal focusing is dominant for larger pattern widths since there is no significant change in thickness as width further increases. The final thickness increases with laser intensity which would be expected for thickness control by spatiotemporal focusing as well as oxygen inhibition.


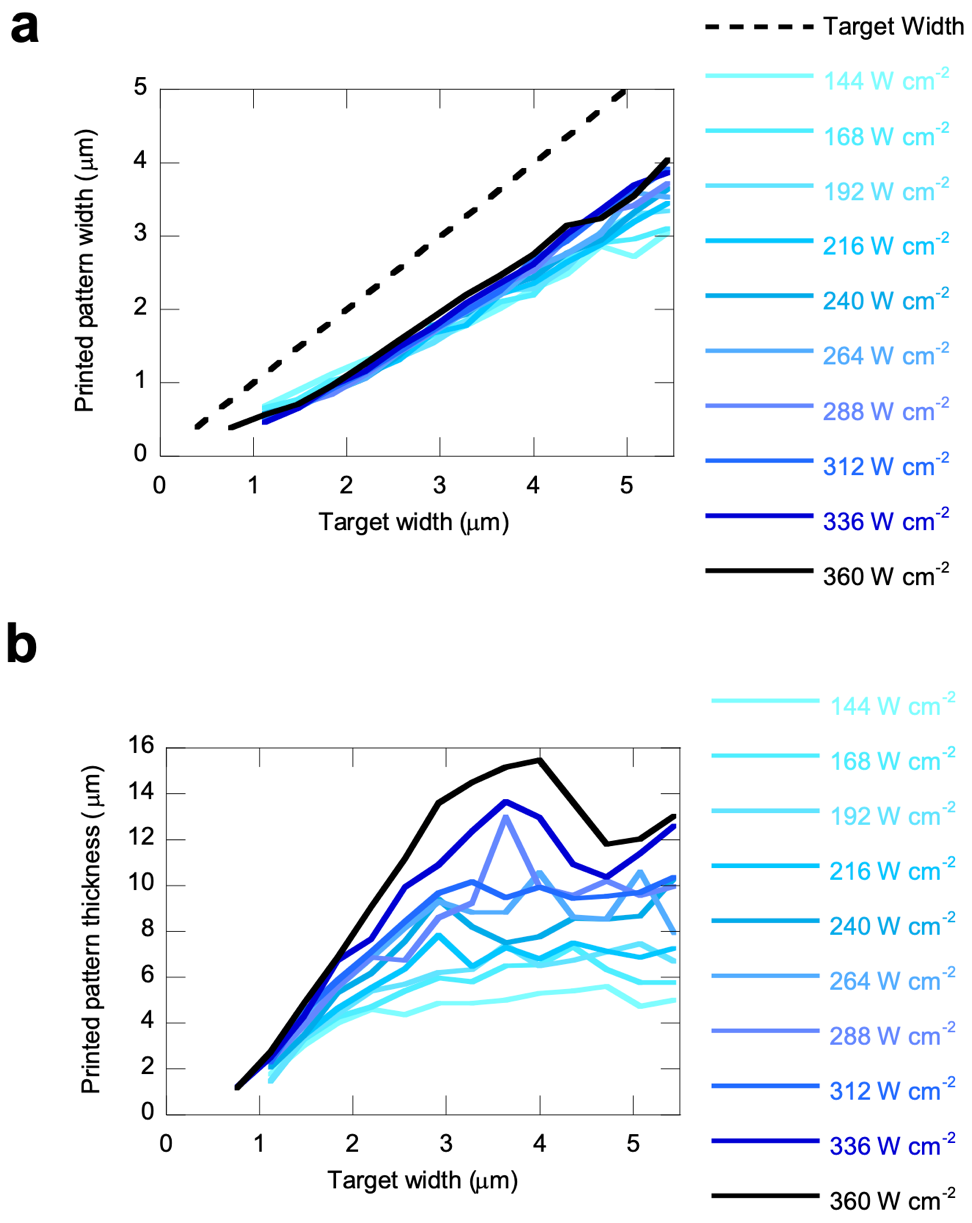


**Fig. S6 Printing results for varying DMD pattern size.** **a** Measured pattern width as a result of projected target width for increasing laser intensities. **b** Measured pattern height as a result of projected target width for increasing laser intensities. All line prints were performed with a stage travel speed of 100 µm s^-1^ and a DMD pattern exposure of 5 ms.

Supplementary Note 3: Projection Printing using Alternate Photoinitiator DETC

The 3D printing capability was tested using an alternate photoresist system composed of 0.25 wt% 7-diethylamino-3-thenoylcoumarin (DETC) in the monomer pentaerythritol triacrylate (PETA). Similar structures as Fig. 5 in the main text were fabricated and are presented in Fig. S7. All structures demonstrate the same structuring capabilities as the BBK photoresist, including smooth surfaces and true 3D features. However, due to a lower photoinitiating efficiency of the DETC resist, printing speed was limited to about 100 µm s^-1^ with laser intensities about 5× higher than required for the BBK resist.


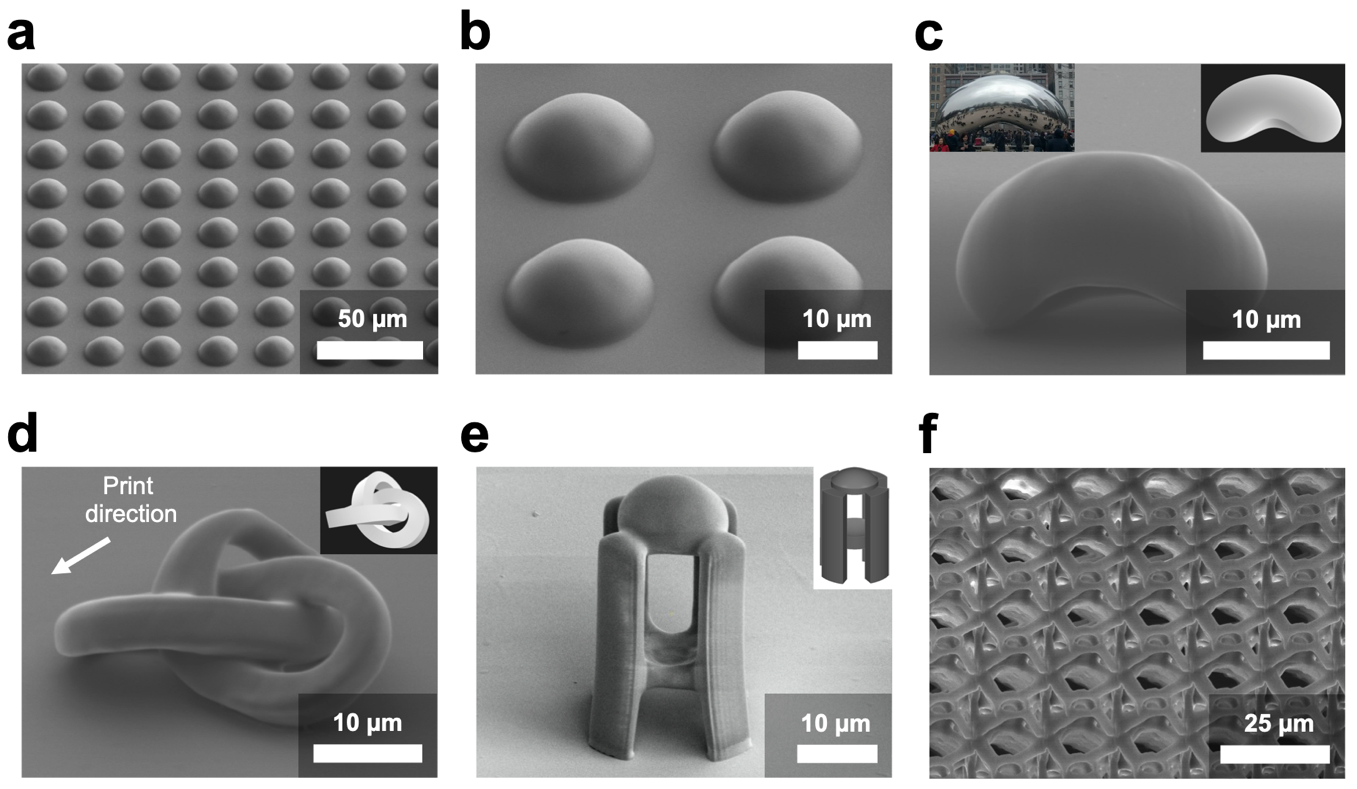


**Fig. S7 3D structures fabricated with DETC containing photoresist.** 3D printed structures using the photoresist composed of 0.25 wt% DETC in the monomer PETA. All structures were fabricated with 100 µm s^-1^ stage print speed and laser intensities between 504 W cm^-2^ and 672 W cm^-2^. **a** Array of hemispheres. **b** Closer image of (**a**). **c** Chicago’s Cloud Gate sculpture. **d** Trefoil knot with square cross-section. **e** Double lens system consisting of a planoconcave lens and a planoconvex lens. **f** 20 × 20 × 20 unit metamaterial-like structure.

Supplementary Note 4: Inspection of the Interior of 3D Printed Structure

A metamaterial-like structure of millimeter scale size, similar to the one shown in Fig. 6, printed using the continuous-layer projection printing system was cut partially in half along the mid-section by femtosecond laser machining. The incident laser direction and cutting path are shown in Fig. S8a. A closer view of the cut cross-section in Fig. S8b shows clear unit cells with unobstructed gaps. This demonstrates that the printing process does not suffer from dose accumulation solidifying the inner volume of the structure and that the printed structure is consistent throughout the entire printed volume.


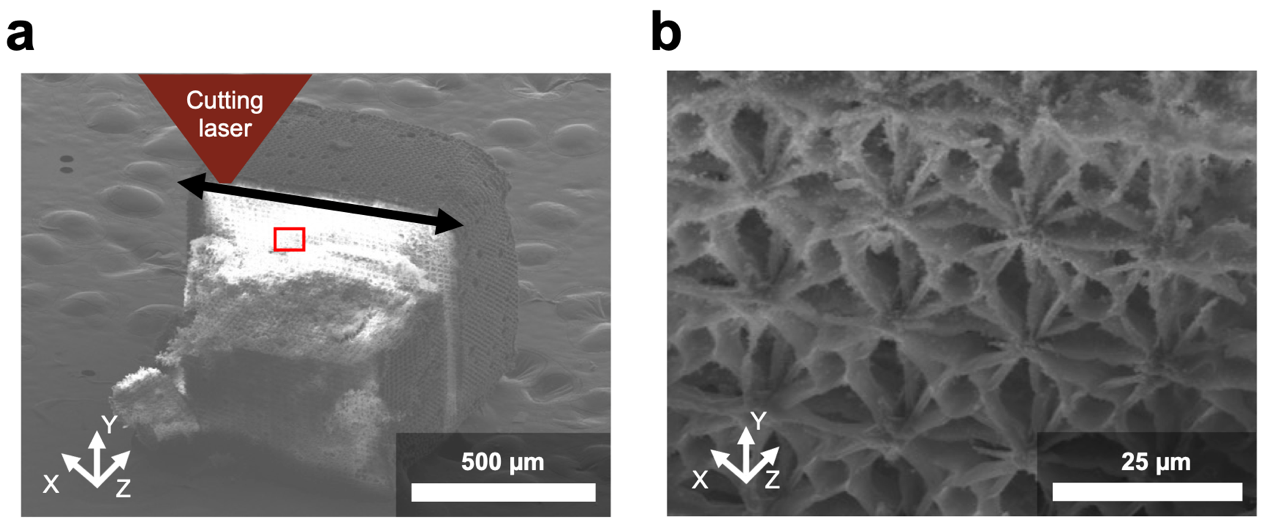


**Fig. S8 Interior view of 3D printed structure using laser cutting.** Millimeter scale metamaterial-like structure fabricated using continuous-layer projection printing partially cut in half by femtosecond laser machining. **a** SEM image of the whole structure. Black arrow indicates cutting path of laser. **b** Enlarged image of cut region indicated by red outline in (**a**). Coordinate system in image indicates coordinates during 3D printing.

Supplementary Note 5: Printing Rate

With the multitude of different 3D printing processes being introduced recently, the new issue has emerged as to how to effectively compare each of them. The two most common methods are volumetric print rate^1,2^ and voxel print rate^3^. For the first, only the physical amount of printed material in a specified time is considered, with no regard to geometry of the structure being fabricated. For the second, the printing process is broken down into its minimum printable feature, the voxel, and then considers how fast the printer can print those voxels. These two metrics are not always directly comparable. For instance, a printer with a very small voxel may be able to achieve a large voxel s^-1^ print rate but their volumetric print rate may be small. On the other hand, a printer with a very small voxel s^-1^ print rate can achieve a very high volumetric print rate with larger voxels. For point printing methods, the voxel rate is significantly more important when one considers the feature size structuring capability of the process. However, for 2D planar printing methods, such as the projection printing scheme in this work, a primary goal is to increase the available print volume. This printing process therefore is in its element when being used to print large area patterns for large volume structuring. Therefore, the volumetric print rate is of more importance. Additionally, one could consider that the projection printing process is capable of rapidly varying its print voxel by the number of pixels activated on the DMD leading to a varying voxel print rate, while the volume print rate would not change. Nonetheless, in order to determine a voxel print rate for the system, a woodpile structure was fabricated at a 1 mm s^-1^ vertical stage speed as shown in Fig. S9. An average linewidth of 380 nm and line height of 2470 nm were measured, leading to a voxel size of 1425 nm (average of lateral and axial dimensions) or ~0.28 µm^3^.


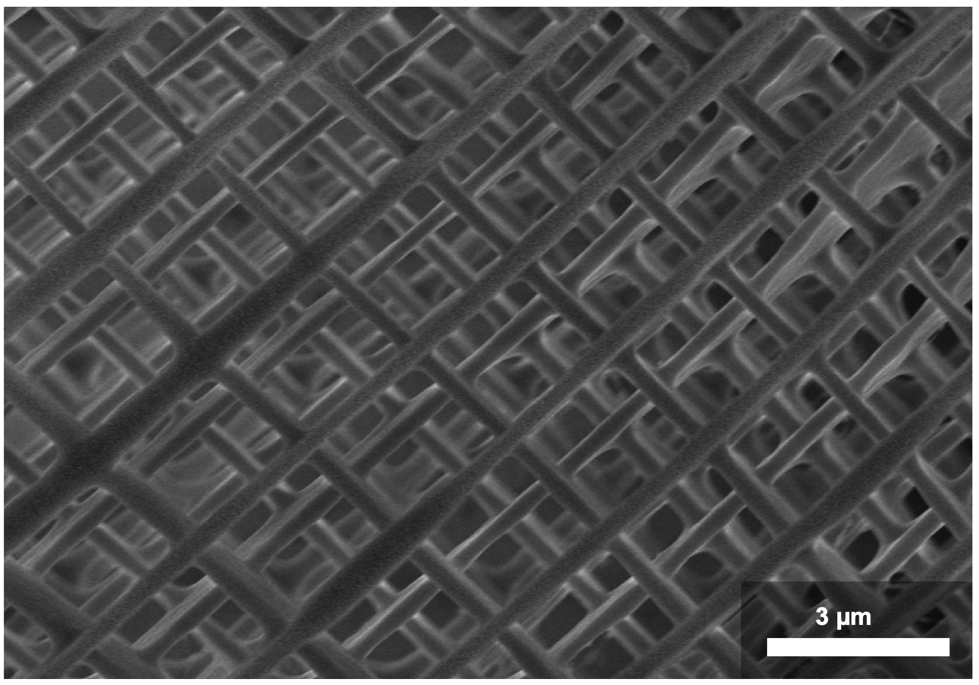


**Fig. S9 Woodpile structure.** Fabricated with 1 mm s^-1^ stage speed and 500 µs pattern exposure time for each layer.

Supplementary Note 6: Fabricating Large Arbitrary 3D Structures

Figure S10 demonstrates the fabrication and print strategy of a large, arbitrary 3D structure. Here, a large structure exhibiting a gradient density has been printed in Figure S10a with the 3D CAD model in Figure S10b. During fabrication of structures larger than the print area of the DMD, multiple smaller print volumes need to be stitched together. In order to overcome the shrinkages of the smaller volumes, a partial overlap of the individual print volumes is implemented. First, the large desired structure is sliced into 2D images. Then each of those images is sectioned into smaller images of the same size as the DMD. Figure S10c provides an example of how it was done for the structure in Figure S10a. Each of the smaller images contains part of the surrounding images, creating the overlap. Figure S10d demonstrates this for a select 4 neighboring images from Figure S10c. Because the DMD system used here is limited to 96 patterns, stitching in the z direction is required as well. This is performed by a similar overlap technique using the above and below layers for each individual volume. This z stitching can more easily be avoided by simply increasing the number of patterns available to be printed in a single z motion.


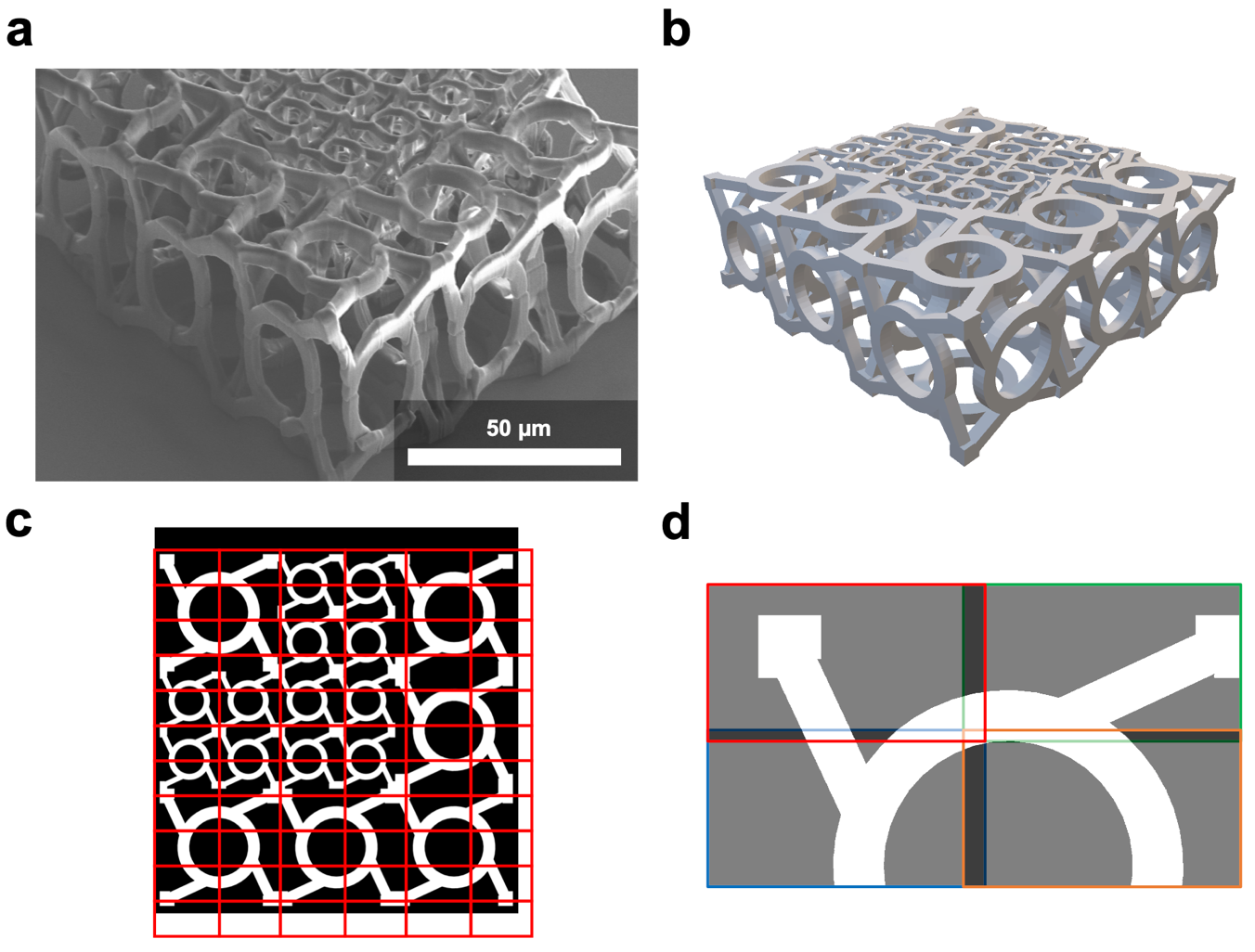


**Fig. S10 Fabrication of large, gradient density structure using overlapping projections. a** Demonstration of fabrication of gradient density architecture structure composed of two different size unit cells. **b** 3D CAD model of structure in (**a**). **c** 2D image of structure with red grid overlayed to indicate the division for individual DMD pattern sizes. **d** Example DMD patterns for 4 sub-volumes of the structure indicating the partial overlapping print scheme. Each pattern is outlined in a separate color to show the overlap.

Supplementary Note 7: Characterization of the BBK Photoinitiator

The nuclear magnetic resonance (NMR) spectroscopy datum of Fig. S11 was acquired using a Bruker AV-III-400-HD NMR spectrometer. The concentration of the molecules in deuterated chloroform was 5%, by weight. The ultraviolet–visible (UV–Vis) light spectroscopy data were obtained using a Cary 60 spectrometer with the wavelength range of 300 nm ≤ λ ≤ 800 nm (Fig. S12).


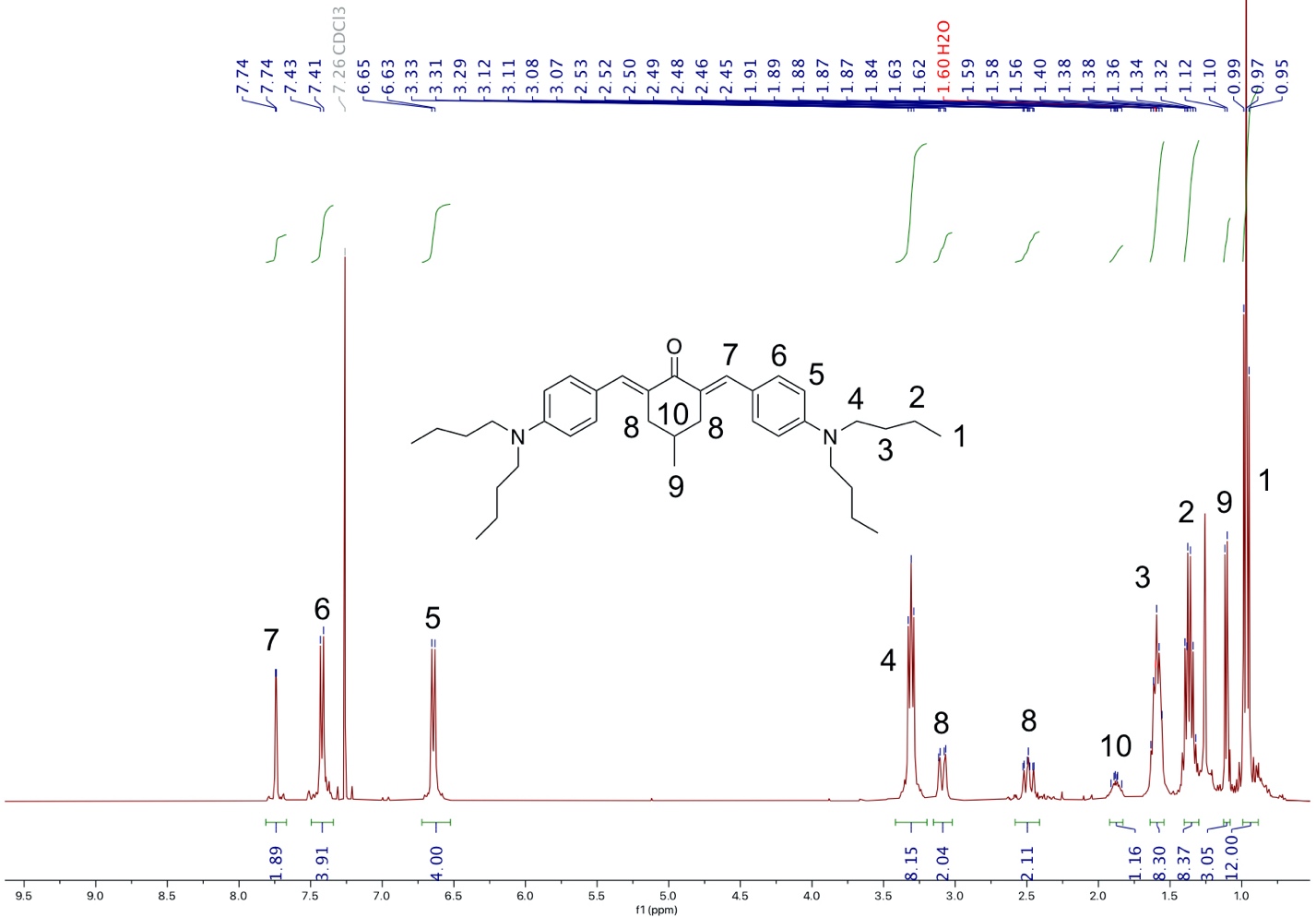


**Fig. S11 ^1^H-NMR spectrum for BBK.**  ^1^H-NMR spectrum of (2E,6E)-2,6-bis (4-(dibutylamino)benzylidene)-4-methylcyclohexanone (BBK) recorded in CDCl_3_.


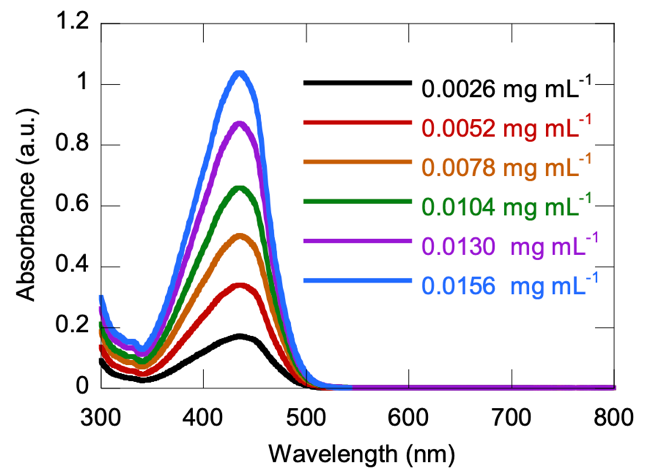


**Fig. S12 UV-Vis spectra of BBK in toluene.** The absorption peak is at 435 nm, and an extinction coefficient of e = 3.6 × 10^4^ M^-1^ cm^-1^ was calculated for the molecule at this wavelength.

References

1. Saha, S. K. *et al.* Scalable submicrometer additive manufacturing. *Science (80-. ).* **366**, 105–109 (2019).

2. Shusteff, M. *et al.* One-step volumetric additive manufacturing of complex polymer structures. *Sci. Adv.* **3**, eaao5496 (2017).

3. Hahn, V. *et al.* Rapid Assembly of Small Materials Building Blocks (Voxels) into Large Functional 3D Metamaterials. *Adv. Funct. Mater.* **30**, 1907795 (2020).
